# Pre-surgical Embolization for Nasal Lobular Capillary Hemangioma: A Case Report

**DOI:** 10.7759/cureus.79502

**Published:** 2025-02-23

**Authors:** Omar Galal, Maryam Jamel, Ayman Elsayed, Aiman Quateen, Sanooj Seyed, Sulaman Magdub

**Affiliations:** 1 Medicine, Khalifa University College of Medicine and Health Sciences, Abu Dhabi, ARE; 2 Radiology, Sheikh Shakhbout Medical City, Abu Dhabi, ARE; 3 Interventional Neuroradiology, Sheikh Shakhbout Medical City, Abu Dhabi, ARE; 4 Otolaryngology - Head and Neck Surgery, Sheikh Shakhbout Medical City, Abu Dhabi, ARE; 5 Pathology and Laboratory Medicine, Sheikh Shakhbout Medical City, Abu Dhabi, ARE

**Keywords:** endoscopic excision, hypervascular nasal lesions, lobular capillary hemangioma, nasal lch, preoperative embolization, sphenopalatine artery

## Abstract

Lobular capillary hemangioma (LCH), a benign vascular lesion, is rare in the nasal cavity but can cause significant symptoms such as epistaxis and nasal obstruction. Preoperative embolization is increasingly used to manage hypervascular nasal LCH, reducing intraoperative bleeding and facilitating complete resection. A 37-year-old woman presented with severe left-sided epistaxis. Imaging revealed a hypervascular left nasal mass (35 x 23 x 18 mm) supplied by the left sphenopalatine artery. Preoperative embolization of the sphenopalatine and posterior superior alveolar arteries was performed, followed by endoscopic excision. Histopathology confirmed LCH. The patient recovered well with no recurrence. Preoperative embolization is critical for large, hypervascular nasal LCH, minimizing intraoperative bleeding and improving surgical outcomes. While smaller lesions may not require embolization, its role in managing larger lesions is well-supported. Imaging and histopathology remain essential for diagnosis and planning. Preoperative embolization is a valuable adjunct in managing nasal LCH, particularly for large or hypervascular lesions. This case highlights its efficacy in ensuring safe and complete resection, emphasizing the importance of a multidisciplinary approach.

## Introduction

Lobular capillary hemangioma (LCH), also known as pyogenic granuloma, is a benign vascular lesion that commonly affects the skin and mucous membranes, particularly in the head and neck region. Although it is frequently found in the oral cavity, nasal LCH is relatively rare, accounting for only 7-29% of head and neck LCH cases [1,2]. Nasal LCH typically presents with symptoms such as epistaxis, nasal obstruction, and occasionally facial pain or headache [3,4]. The etiology of LCH remains unclear, but factors such as trauma, hormonal changes (e.g., pregnancy or oral contraceptive use), and local irritation have been implicated in its development [5,6].

Diagnosis of nasal LCH is often challenging due to its nonspecific symptoms and resemblance to other hypervascular nasal lesions, such as angiofibroma, hemangiopericytoma, and angiosarcoma [1,3]. Imaging studies, including computed tomography (CT) and magnetic resonance imaging (MRI), are essential for assessing the extent of the lesion and its vascular supply, which is critical for surgical planning [7,8]. Histopathological examination remains the gold standard for definitive diagnosis, revealing characteristic lobular arrangements of capillaries with surrounding fibromyxoid stroma [9].

The standard treatment for nasal LCH is complete surgical excision, often performed endoscopically to minimize morbidity [3,4]. However, due to the highly vascular nature of these lesions, preoperative embolization has been advocated to reduce intraoperative bleeding and facilitate complete resection [1,6]. This case report highlights the efficacy of preoperative embolization in managing a large nasal LCH, emphasizing its role in reducing surgical complications and ensuring successful outcomes.

## Case presentation

A previously healthy 37-year-old woman presented to the emergency department with severe left-sided epistaxis. The bleeding was initially managed with nasal packing, and the patient was hemodynamically stable but had a low hemoglobin level of 116 g/L. She denied any history of trauma, bleeding disorders, or use of hormonal medications. Apart from a headache, likely secondary to the epistaxis, she had no other symptoms.

**Figure 1:**
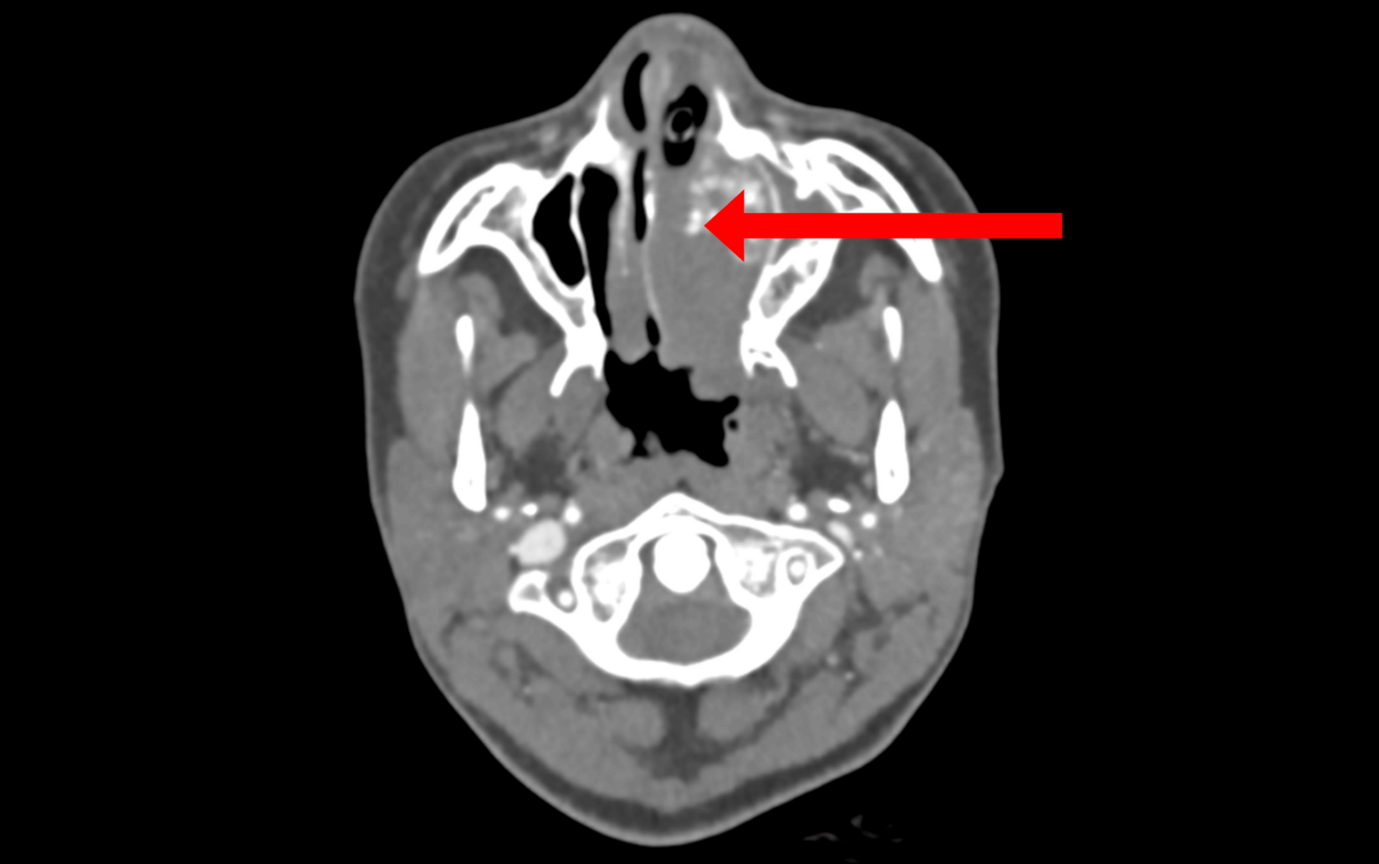
CT shows a well-circumscribed soft-tissue mass in the left nasal cavity with outward bowing of the medial wall of the maxillary sinus (red arrow).

**Figure 2:**
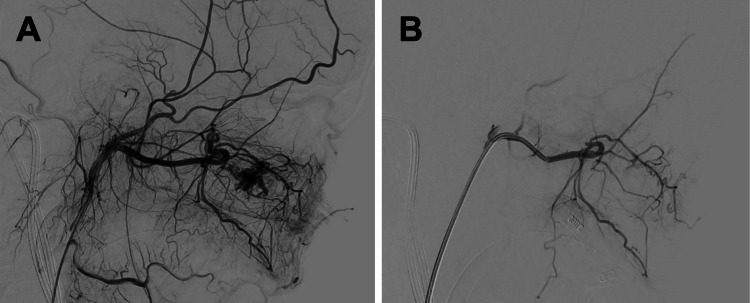
(A) Pre-embolization: MR angiography shows a vascular blush within the lesion. (B) Post-embolization: MR angiography shows reduced vascular blush, suggesting successful reduction of blood flow within the lesion.

Upon removal of the nasal packing, a lateral nasal wall mass was identified during endoscopic examination. CT angiography of the head and neck revealed a highly vascular left-sided nasal mass measuring approximately 35 x 23 x 18 mm (Figure 1). The mass compressed the nasal septum, extended into the left postnasal space, and encroached on the anterior aspect of the nasopharyngeal airway. It was pedicled at the inferior aspect of the inferior turbinate. The left sphenopalatine artery was notably prominent, suggesting it to be the primary feeding vessel. MRI and MR angiography further confirmed the vascular blush within the lesion, consistent with a hypervascular tumor supplied by branches of the left sphenopalatine artery (Figure 2a).

**Figure 3:**
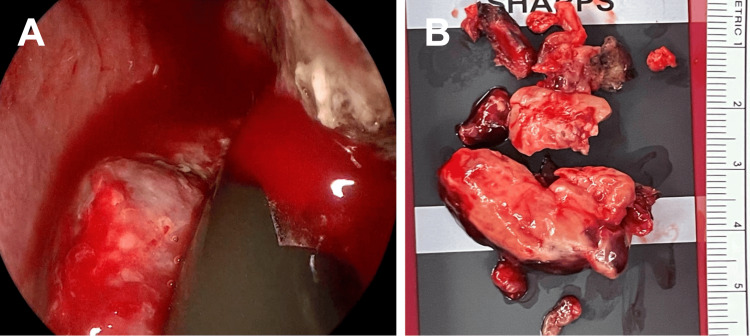
(A) Endoscopy revealed a large vascular mass pedicled at the inferior aspect of the inferior turbinate. (B) Gross appearance of the excised mass, measuring approximately 5 x 2 cm.

**Figure 4:**
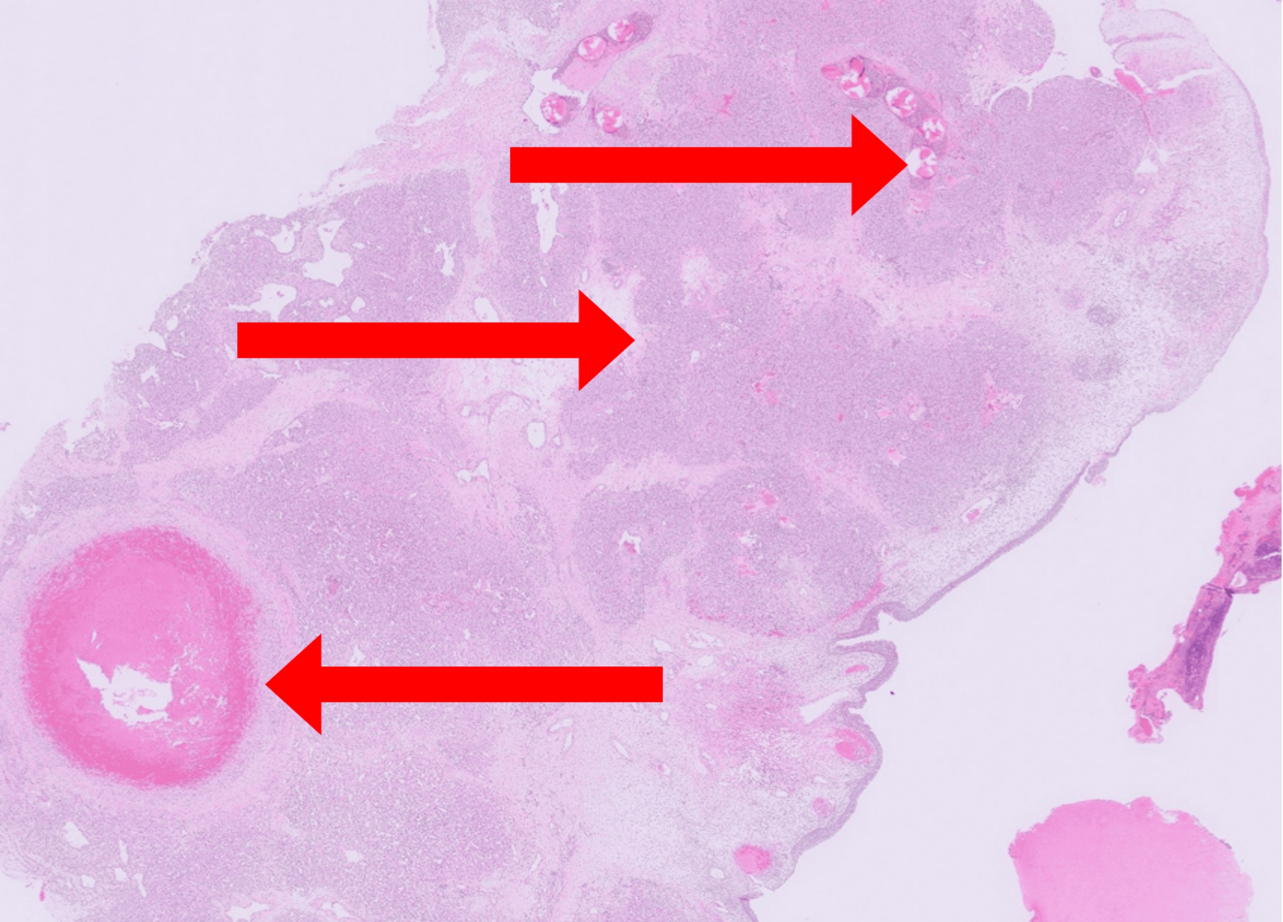
Histopathology of the lesion revealed lobulated capillary proliferation with associated feeding blood vessels, suggesting LCH (red arrows). This is a low-magnification (4x) histopathology image stained with H&E. LCH: lobular capillary hemangioma; H&E: hematoxylin and eosin

Given the vascular nature of the lesion, preoperative embolization of the left sphenopalatine artery and the posterior superior alveolar artery was performed using Embosphere particles (300-400 microns). The procedure successfully reduced the vascular blush (Figure 2b), and the patient remained hemodynamically stable throughout, with minimal blood loss. Following embolization, the mass was excised endoscopically, measuring approximately 5 x 2 cm in total (Figure 3). Histopathological examination revealed lobulated capillary proliferation with associated feeding blood vessels, confirming the diagnosis of LCH (Figure 4).

Postoperatively, the patient recovered well, with no further episodes of epistaxis or nasal obstruction. Follow-up examinations confirmed the complete resolution of symptoms, and the patient remained asymptomatic at subsequent visits.

## Discussion

This case highlights the importance of preoperative embolization in the management of nasal LCH, particularly for large, hypervascular lesions. Nasal LCH is a benign but highly vascular tumor that can cause significant bleeding during surgical manipulation, making preoperative vascular control essential [1,6]. In this case, embolization of the left sphenopalatine artery and posterior superior alveolar artery significantly reduced the vascularity of the lesion, facilitating a safe and complete resection with minimal blood loss. While the exact amount of blood loss was not quantified, the preoperative mean arterial blood pressure was 96 mmHg and the postoperative mean arterial blood pressure was 82 mmHg, indicating relative hemodynamic stability during the procedure. 

The efficacy of preoperative embolization in nasal LCH has been well-documented in the literature. For instance, Albesher et al. (2023) reported a case of a large nasal LCH where preoperative embolization reduced intraoperative bleeding and allowed for successful endoscopic excision [6]. Similarly, Tamaki et al. (2017) described two cases of nasal LCH where preoperative embolization or sphenopalatine artery ligation was crucial in managing intraoperative hemorrhage [1]. In both cases, the authors emphasized that preoperative vascular control significantly reduced blood loss and improved surgical outcomes, aligning with the findings in our case. Furthermore, Chi et al. (2014) reported a series of 15 patients with nasal LCH, all of whom underwent surgical excision without preoperative embolization. While none of their patients experienced significant bleeding, the authors noted that larger lesions might benefit from preoperative vascular control to minimize surgical risks [4].

The debate over the necessity of preoperative embolization in nasal LCH is ongoing. Some authors argue that embolization may not be required for smaller lesions, as endoscopic excision alone can be performed safely with minimal bleeding. Puxeddu et al. (2006) reported a series of 40 patients with nasal LCH, most of whom underwent endoscopic excision without preoperative embolization. However, they noted that larger lesions, especially those involving the lateral nasal wall, may benefit from preoperative vascular control to reduce bleeding and improve surgical outcomes [3]. This is consistent with our case, where the large size (35 x 23 x 18 mm) and hypervascular nature of the lesion necessitated embolization. The prominent left sphenopalatine artery identified on imaging further underscored the need for preoperative vascular control, as this vessel was the primary source of blood supply to the tumor.

The role of imaging in diagnosing and planning the management of nasal LCH cannot be overstated. CT and MRI are invaluable for assessing the extent of the lesion, identifying feeding vessels, and differentiating LCH from other hypervascular nasal tumors [6,7,10]. In our case, CT angiography and MR angiography provided critical information about the vascular supply of the lesion, guiding the decision to perform preoperative embolization. Histopathological examination remains the definitive diagnostic tool for nasal LCH, revealing characteristic lobular arrangements of capillaries with surrounding fibromyxoid stroma [2]. In our case, the histopathological findings were consistent with LCH, confirming the diagnosis and ruling out other vascular lesions such as angiofibroma or hemangiopericytoma.

The management of nasal LCH has evolved significantly over the years, with endoscopic surgery now considered the gold standard for treatment [3,4]. Endoscopic excision offers several advantages, including improved visualization, reduced morbidity, and faster recovery times. However, the highly vascular nature of LCH can pose challenges during surgery, particularly for larger lesions. Preoperative embolization has emerged as a valuable adjunct to endoscopic surgery, reducing intraoperative bleeding and facilitating complete resection [1,6]. In our case, embolization of the left sphenopalatine artery and posterior superior alveolar artery was performed successfully, resulting in minimal blood loss and a smooth surgical procedure. Embolic microsphere particles of size greater than 150 microns, such as the one used in this case, have been shown to efficiently penetrate the tumor's capillary bed while remaining safe [11]. Although preoperative embolization may not be necessary for all cases of nasal LCH, it should be strongly considered for large lesions or those with significant vascularity. This approach minimizes surgical risks and improves patient outcomes, as demonstrated in this case.

## Conclusions

This case report underscores the efficacy of preoperative embolization in the management of nasal LCH, particularly for large, hypervascular lesions. By reducing intraoperative bleeding and facilitating complete resection, embolization plays a critical role in ensuring successful surgical outcomes. Imaging studies, including CT and MRI, are essential for preoperative planning, while histopathological examination remains the gold standard for diagnosis. Although preoperative embolization may not be necessary for all cases of nasal LCH, it should be strongly considered for large lesions or those with significant vascularity. However, embolization has limitations, including potential risks such as vascular injury, incomplete embolization, and the need for specialized interventional radiology expertise. This approach minimizes surgical risks and improves patient outcomes, as demonstrated in this case.
